# Molecular Cloning, Characterization, and Functional Analysis of Acetyl-CoA *C*-Acetyltransferase and Mevalonate Kinase Genes Involved in Terpene Trilactone Biosynthesis from *Ginkgo biloba*

**DOI:** 10.3390/molecules22010074

**Published:** 2017-01-02

**Authors:** Qiangwen Chen, Jiaping Yan, Xiangxiang Meng, Feng Xu, Weiwei Zhang, Yongling Liao, Jinwang Qu

**Affiliations:** College of Horticulture and Gardening, Yangtze University, Jingzhou 434025, Hubei, China; chenqwx@foxmail.com (Q.C.); xiaolinyingxue@163.com (J.Y.); 15927855989@163.com (X.M.); zww8312@163.com (W.Z.); liaoyongling@yeah.net (Y.L.); qujinwang@163.com (J.Q.)

**Keywords:** *Ginkgo biloba*, *GbAACT*, *GbMVK*, SA, MeJA, functional complementation

## Abstract

Ginkgolides and bilobalide, collectively termed terpene trilactones (TTLs), are terpenoids that form the main active substance of *Ginkgo biloba*. Terpenoids in the mevalonate (MVA) biosynthetic pathway include acetyl-CoA *C*-acetyltransferase (AACT) and mevalonate kinase (MVK) as core enzymes. In this study, two full-length (cDNAs) encoding AACT (*GbAACT*, GenBank Accession No. KX904942) and MVK (*GbMVK*, GenBank Accession No. KX904944) were cloned from *G. biloba*. The deduced GbAACT and GbMVK proteins contain 404 and 396 amino acids with the corresponding open-reading frame (ORF) sizes of 1215 bp and 1194 bp, respectively. Tissue expression pattern analysis revealed that *GbAACT* was highly expressed in ginkgo fruits and leaves, and *GbMVK* was highly expressed in leaves and roots. The functional complementation of *GbAACT* in AACT-deficient *Saccharomyces cerevisiae* strain *Δerg10* and *GbMVK* in MVK-deficient strain *Δerg12* confirmed that *GbAACT* mediated the conversion of mevalonate acetyl-CoA to acetoacetyl-CoA and *GbMVK* mediated the conversion of mevalonate to mevalonate phosphate. This observation indicated that *GbAACT* and *GbMVK* are functional genes in the cytosolic mevalonate (MVA) biosynthesis pathway. After *G. biloba* seedlings were treated with methyl jasmonate and salicylic acid, the expression levels of *GbAACT* and *GbMVK* increased, and TTL production was enhanced. The cloning, characterization, expression and functional analysis of *GbAACT* and *GbMVK* will be helpful to understand more about the role of these two genes involved in TTL biosynthesis.

## 1. Introduction

*Gingko biloba* L., which dates back to more than 200 million years, is the only surviving member of ginkgophyta in gymnosperm family and considered a “living fossil” [1]. Ginkgo leaf extracts are used to treat cardiovascular and cerebrovascular diseases because of their highly specific and potent platelet-activating factor receptor antagonists [2]. *G. biloba* contains terpene trilactones (TTLs), such as diterpenoid ginkgolides and sesquiterpenoid bilobalide, as the main bioactive components [3]. TTLs in *G. biloba* possess unique biological properties and promote high activities of anti-platelet-activation factors [4]. TTLs also function as a selective glycine receptor and participate as the main bioactive substance in *G*. *biloba* [5]. However, there are many difficulties with respect to supply of ginkgo leaves and chemical synthesis is far from of being applicable for commercial-scale production. Different biotechnological strategies to improve TTL production have been used, including screening and selection of in vitro ginkgo cultures, cell differentiation levels of these cultures, and optimization of culture conditions, feeding the elicitation strategies [6]. However, until now, the yields obtained from cell cultures have been low and new strategies such as the use of key genes for increasing TTL production by genetic engineering are imperative.

**Figure 1:**
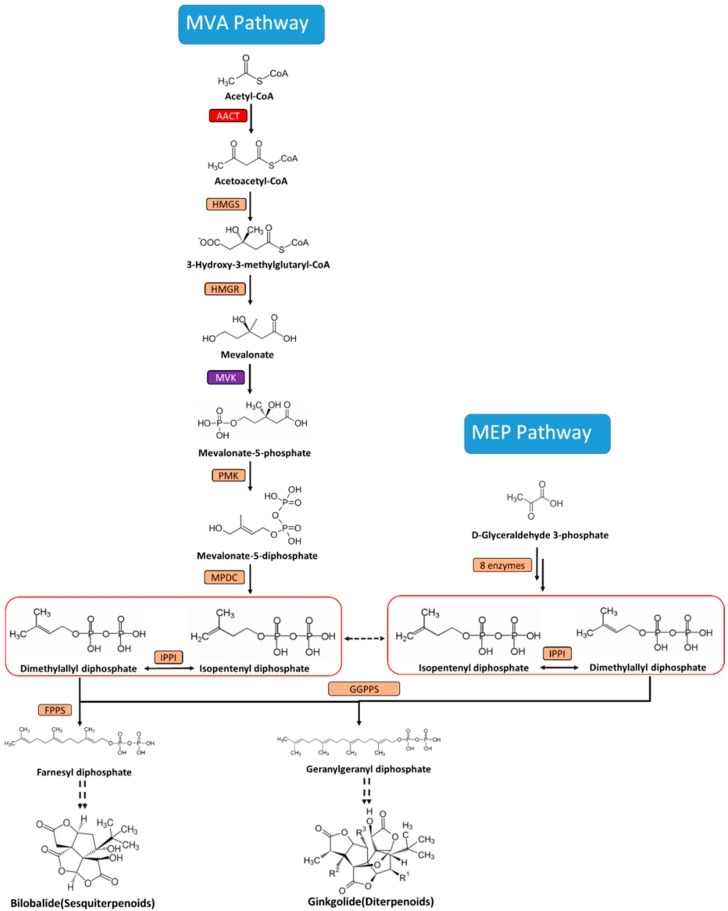
The biosynthetic pathway of ginkgolides and bilobalide in *Ginkgo biloba*. MEP: methylerythritol 4-phosphate; MVA: mevalonate; AACT: acetyl-CoA *C*-acetyltransferase; HMGS: 3-Hydroxy-3-methylglutaryl-CoA synthase; HMGR: 3-Hydroxy-3-methylglutaryl-CoA reductase; MVK: mevalonate kinase; PMK: Phosphomevalonate Kinase; MPDC: Diphospho-MVA decarboxylase; IPPI: Isopentenyl diphosphate isomerase; FPPS: Farnesyl diphosphate synthase; GGPPS: Geranylgeranyl diphosphate synthase.

Until recently, the terpenoid origin of the plant was considered to have two involved precursors—isopentenyl pyrophosphate (IPP) and its isomer dimethylallyl pyrophosphate (DMAPP). IPP and DMAPP are synthesized via two indenpendent isoprenoid pathways: the cytosolic mevalonate (MVA) pathway and the plastidial methylerythritol 4-phosphate (MEP) pathway (Figure 1). In the MVA pathway, three acetyl-CoA molecules are converted into isopentenyl diphosphate (IPP), and this process is catalyzed by seven enzymes. In the MEP pathway, IPP is similarly produced, and an IPP isomer called dimethylallyl pyrophosphate is formed from pyruvate and d-glyceraldehyde-3-phosphate via eight enzymatic reactions. Generally, the main MVA-derived isoprenoid end-products are sesquiterpenoids and sterols, whereas the monoterpenoids and diterpenoids are derived from the MEP pathway [7]. However, an IPP crosstalk exists between plastids and cytosol, and this observation suggests that the MVA pathway also contributes to TTL biosynthesis [8,9]. A few of genes involved in the MVA and MEP pathway have been cloned and identified in *G. biloba*, such as mevalonate diphosphate decarboxylase (MVD) [10,11], 3-hydroxy-3-methylglutaryl coenzyme A reductase (HMGR) [12], 1-deoxy-d-xylulose-5-phosphate synthase (DXS) [13], 1-deoxy-d-xylulose-5-phosphate reductoisomerase (DXR) [14], 4-(cytidine-5′-diphospho)-2-*C*-methyl-d-erythritol kinase (CMK) [15], 2-*C*-methyl-d-erythritol-2,4-cyclodiphosphate synthase (MECS) [16,17], 1-hydroxy-2-methyl-2-(*E*)-butenyl-4-diphosphate synthase (HDS) [18], and 1-hydroxy-2-methyl-2-(*E*)-butenyl-4-diphosphate reductase (IDS) [19]. TTL content in *G. biloba* could be enhanced by upregulating the transcript level of some of these genes [11,15,19,20]. Thus, cloning and characterization the genes involved in TTL biosynthesis could provide good candidates for metabolic engineering to increase TTL production in *G. biloba*.

In the MVA pathway, acetyl-CoA *C*-acetyltransferase (AACT) is the first enzyme that catalyzes the conversion of acetyl-CoA into acetoacetyl-CoA. Mevalonate kinase (MVK) is the fourth enzyme that catalyzes the conversion of MVA into mevalonate-5-phosphate, which undergoes an enzymatic reaction catalyzed by phospho-mevalonate kinase to generate mevalonate-5-diphosphate. Mevalonate-5-diphosphate is then transformed into isopentenyl diphosphate, as catalyzed by MVD. *AACT* and *MVK* genes have been described in plants; while reports on these genes are few, what we do know is that *AACT* is found in *Elaeis guineensis* Jacq [21], *Ganoderma lucidum* [22], and *Bacopa monnieri* [23], and *MVK* is found in *Eucommia ulmoides* [24] and *Hevea brasiliensis* [25]. However, cloning and characterization of *AACT* and *MVK* from *G. biloba* have not been reported in the literature. In this study, two novel cDNAs of *AACT* and *MVK* were cloned and characterized from *G. biloba*. Yeast complementation assays were conducted to identify the function of these genes. The expression patterns of *GbAACT* and *GbMVK* in various tissues, including roots, stems, leaves, fruits, male and female flowers were also examined to describe TTL synthesis. In addition, our previous work showed that the transcripts of *HMGR* [26] and *MVD* [11] genes involved in the MVA pathway are positively responsive to methyl jasmonate (MeJA) and salicylic acid (SA) treatments in ginkgo. Therefore, the expression profiles of *GbAACT* and *GbMVK* as well as TTL contents under the induction by MeJA and SA were also investigated, which will facilitate future work to map and regulate these important steps involved in TTL biosynthetic pathway at the level of molecular genetics.

## 2. Results and Discussion

### 2.1. Isolation and Characterization of the cDNA of GbAACT and GbMVK

The full-length cDNA sequences of *GbAACT* and *GbMVK* were cloned through RT-PCR (Reverse transcription polymerase chain reaction). Using total RNA isolated from the young leaves of *G. biloba*, a 2028-bp and 2057-bp fragment was amplified through RT-PCR. After aligating these genes into pMD19-T vector and sequencing, the results of sequence analysis showed that *GbAACT* contained an open-reading frame (ORF) of 1215 bp and that *GbMVK* contained an ORF of 1194 bp. The *GbAACT* encoded a protein containing 404 amino acids with a 6.33 isoeletric point, and the calculated molecular weight was approximately 41.5 kDa. Meanwhile, *GbMVK* encoded a protein containing 397 amino acids with a 5.71 isoeletric point, and the calculated molecular weight was approximately 42 kDa. A BLASTn search of *GbAACT* and *GbMVK* with other plant species showed that *GbAACT* and *GbMVK* are highly homologous to *AACT* genes and *MVK* genes from other plant species. Therefore, these genes were designated as *GbAACT* (GenBank accession No. KX904942) and *GbMVK* (GenBank accession No. KX904944).

### 2.2. Bioinformatics Analysis of the Deduced GbAACT and GbMVK Protein

A BLASTp search against nonredundant (nr) protein in GenBank database (https://blast.ncbi.nlm.nih.gov) showed that the polypeptide sequence of GbAACT had 78%–81% homology with AACTs of many other plant species, such as *Nelumbo nucifera* (NnAACT, Identity: 81%, Accession No. XP_010252788.1), *Amborella trichopoda* (AtAACT, Identity: 80%, Accession No. XP_011628271.1), *Hevea brasiliensis* (HbAACT, Identity: 81%, Accession No. AFJ74323.1), *Ricinus communis* (RcAACT, Identity: 81%, Accession No. XP_015577103.1), *Morus alba* (MaAACT, Identity: 80%, Accession No. ALD84318.1), and *Euphorbia helioscopia* (EhAACT, Identity: 78%, Accession No. ALC76524.1). The deduced amino acid sequence of GbMVK had a high degree of homology, from 61% to 63% with the MVK protein of other plant species, such as *Prunus mume* (PmMVK, Identity: 62%, Accession No. XP_008246488.1), *Pyrus* × *bretschneideri* (PbMVK, Identity: 63%, Accession No. XP_009349394.1), *Jatropha curcas* (JcMVK, Identity: 62%, Accession No. XP_012089078.1), *Ziziphus jujuba* (ZjMVK, Identity: 62%, Accession No. XP_015875214.1), and *Hevea brasiliensis* (HbMVK, Identity: 63%, Accession No. AIO11226.1).

**Figure 2:**
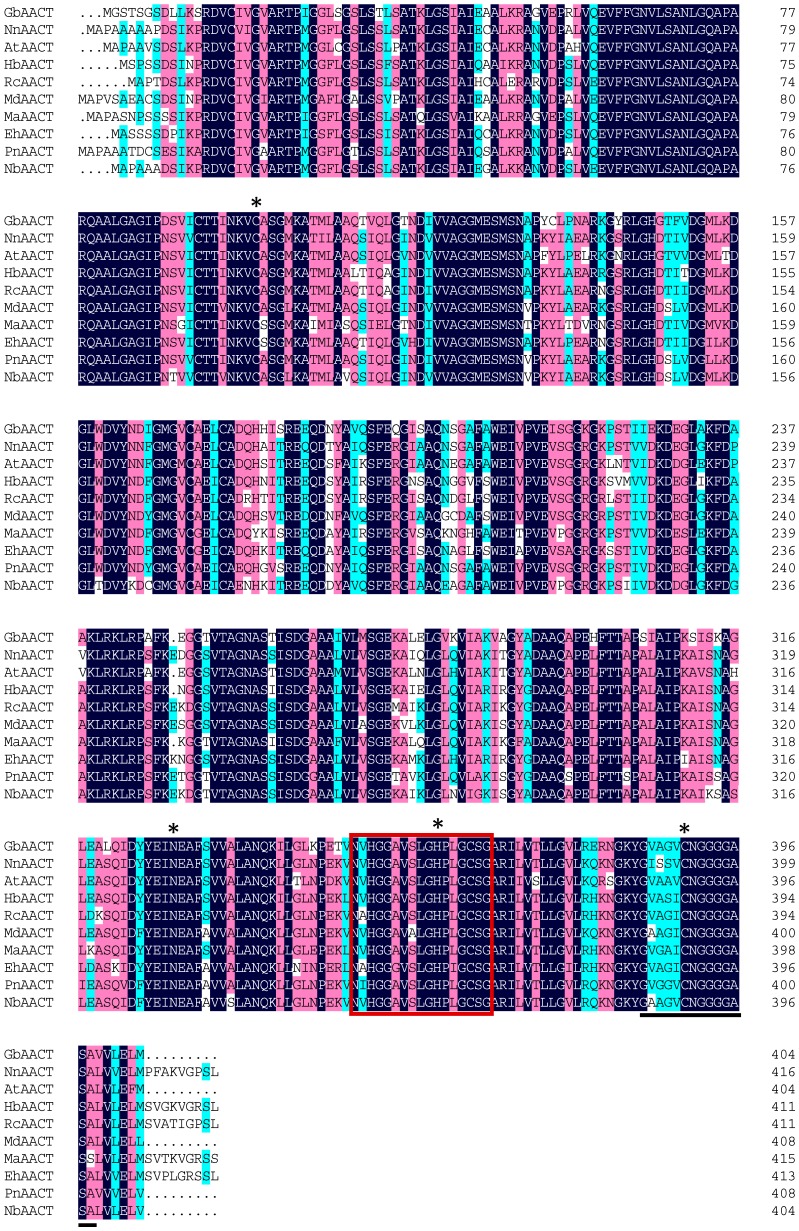
Amino acid sequence multiple alignments of GbAACT with other plant AACTs from *Nelumbo nucifera* (NnAACT, Accestion No. XP_010267976.1), *Amborella trichopoda* (AtAACT, Accestion No. XP_011628272.1), *Hevea brasiliensis* (HbAACT, Accestion No. AFJ74323.1), *Malus domestica* (MdAACT, Accestion No. XP_008365553.1), *Morus alba* (MaAACT, Accestion No. ALD84318.1), *Euphorbia helioscopia* (EhAACT, Accestion No. ALC76524.1), *Panax notoginseng* (PnAACT, Accestion No. AIK21787.1), *Nicotiana benthamiana* (NbAACT, Accestion No. BAR94039.1). Dark blue: identity = 100%; red: 75% ≤ identity < 100%; light blue: 50% ≤ identity < 75%. The active sites of amino acid residues are marked with asterisk, Conserved domain (NVHGGAVSIGHPIGCSG) is marked with red frame, thiolase II active sites (GVAGVCNGGGGASA) are underlined.

Multiple alignments of GbAACT with AACTs from other plants indicated that the plant AACTs were the most similar (Figure 2). The online InterPro result showed that the function of GbAACT harbors the activity of thiolase II, and the structure of GbAACT monomer contains three domains, including thiolase-like domain (14–280), N-terminal (15–273), and C-terminal (282–402). Based on the differences in catalytic activities, the thiolases are of two types: thiolase I (acetyl-CoA *C*-acyltransferase) and thiolase II (acetyl-CoA *C*-acetyltransferase). Thiolase I is a degradative thiolase, and thiolase II is a synthetic thiolase [27]. Residues of two cystines, one histidine, and one asparagine are present in GbAACT, which are highly conserved in AACT among the thiolases from different sources, and are important for catalytic activity (Figure 2, marked with “asterisk”) [28,29]. One highly conserved domain (NVHGGAVSIGHPIGCSG) at the C-terminal end is also present. The thiolase II active site GVAGVCNGGGGASA at the last position is specific for AACT [30]. This evidence confirms the similarity function of GbAACT to AACTs from other plants.

**Figure 3:**
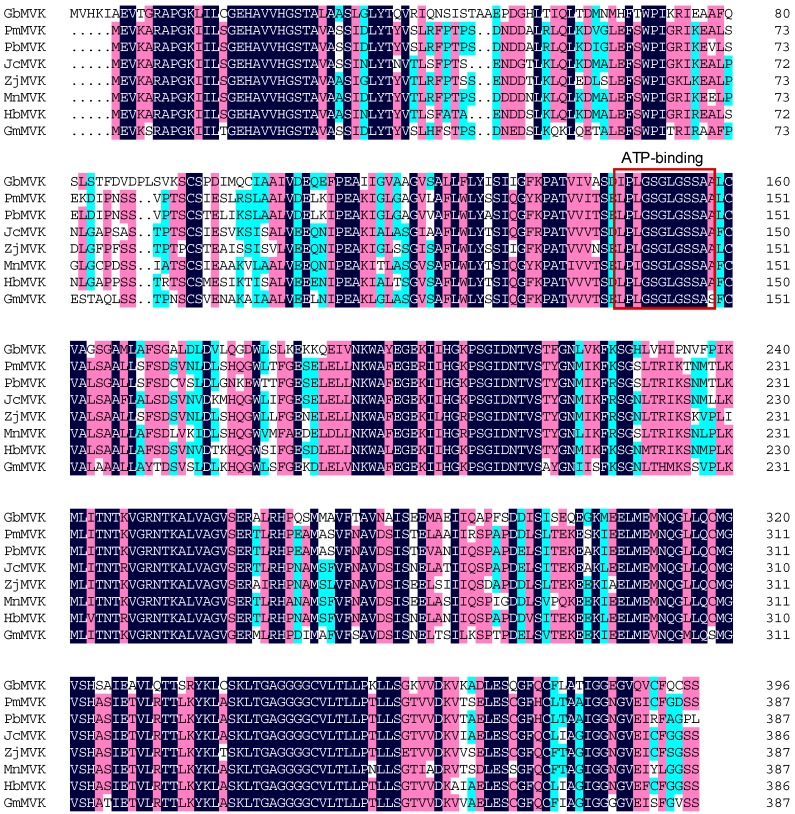
Multiple alignments of GbMVK with MVK proteins from *Prunus mume* (PmMVK, Accestion No. XP_008246488.1), *Pyrus* × *bretschneideri* (PbMVK, Accestion No. XP_009349394.1), *Jatropha curcas* (JcMVK, Accestion No. XP_012089078.1), *Ziziphus jujuba* (ZjMVK, Accestion No. XP_015875214.1), *Morus notabilis* (MnMVK, Accestion No. XP_010105842.1), *Hevea brasiliensis* (HbMVK, Accestion No. AIO11226.1), *Glycine max* (GmMVK, Accestion No. NP_001276217.1). Dark blue: identity = 100%; red: 75% ≤ identity < 100%; light blue: 50% ≤ identity < 75%.

Sequence alignment using Vector NTI 11.5.1 showed that the predicted GbMVK shared a high level of identity with MVKs from other plant species, implying that GbMVK harbors the activity of mevalonate kinase. The structure of GbMVK monomer contains ribosomal protein S5 2-type (8–230), GHMP kinase N-terminal domain (140–220), and GHMP kinase C-terminal domain (236–390). Ile_146_–Ala_157_ is an ATP-binding conserved site (Figure 3, marked with red box), and these findings indicated that GbMVK has a similar catalytic function to other plant MVKs.

### 2.3. Molecular Evolution Analysis

**Figure 4:**
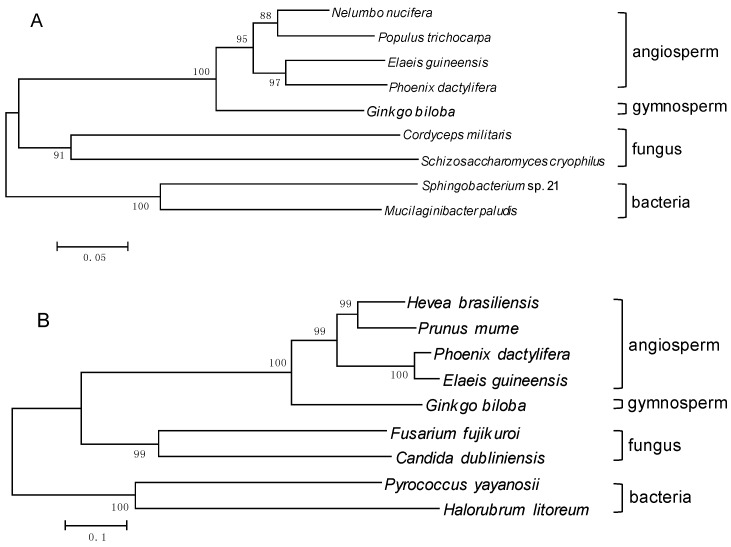
Phylogenetic analysis of the amino acid sequences of AACTs (**A**) and MVKs (**B**). The neighbor-joining phylogenetic trees were constructed using the bootstrap method on MEGA 6.01 and the number of Bootstrap replications was 1000. The following are the protein sequences used in these trees: (**A**) AACT: *Populus trichocarpa* (XP_002308755.1), *Nelumbo nucifera* (XP_010252788.1), *Elaeis guineensis* (XP_010936771.1), *Phoenix dactylifera* (XP_008811276.1), *Schizosaccharomyces cryophilus* (XP_013022052.1), *Cordyceps militaris* (XP_006672862.1), *Mucilaginibacter paludis* (WP_040627967.1), *Sphingobacterium* sp. 21 (WP_013665750.1); (**B**) MVK: *Hevea brasiliensis* (ALR72881.1), *Prunus mume* (XP_008246488.1), *Elaeis guineensis* (XP_010906879.1), *Phoenix dactylifera* (XP_008790991.1), *Fusarium fujikuroi* (KLO96633.1), *Candida dubliniensis* CD36 (XP_002417536.1), *Pyrococcus yayanosii* (WP_013905018.1), *Halorubrum litoreum* (WP_008367863.1).

To investigate the evolutionary relations among deduced GbAACT with other AACTs, and among GbMVK with other MVKs from angiosperm, gymnosperm, fungus, and bacteria, two phylogenetic trees were constructed using the neighbor-joining (NJ) method with *p*-distance. Apparently, the bootstrap value is much high for each interior branch with a high identity (>90%). Therefore, the constructed phylogenetic trees of AACTs and MVKs are reliable. As shown in Figure 4A, AACTs from different species seemed to evolve into different groups, with fungus as an ancient group. Then the bacteria group containing AACTs diverged from *Sphingobacterium* and *Mucilaginibacter paludis*. The plant AACT group diverged later than the fungus and bacteria. Among the plant group, the GbAACT diverged a little earlier than AACT from other angiosperm plant species in the phylogenetic tree, which coincided with the evolutional position of *G. biloba* as the most ancient among gymnosperm plant species. As shown in Figure 4B, MVKs from different species evolved vertically from a common ancestor. The MVKs from different species evolved into various groups, with bacteria as an ancient group, and then the fungus group containing MVKs diverged from *Fusarium fujikuroi* and *Candida dubliniensis*. The plant species divided into angiosperm and gymnosperm species, and the GbMVK diverged earlier than MVK from other plant species. These results correspond with the fact that *G. biloba* is the most ancient among gymnosperm plant species. Given that both the AACTs and MVKs can be found in fungus, bacteria, and higher plants, the bioinformatics analysis also indicated that GbAACT was a plant AACT protein with AACT activity and that GbMVK was a plant MVK protein with GHMP kinase activity. Both *GbAACT* and *GbMVK* can also be inferred to be a class of highly conservative ancient genes.

### 2.4. Functional Complementation of GbAACT and GbMVK in Saccharomyces Cerevisiae

**Figure 5:**
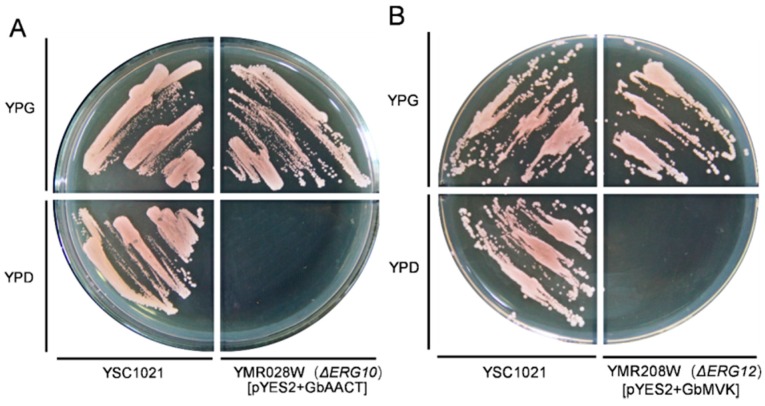
Functional complementation for the growth of haploid disrupted strains *Δerg10* complemented with *GbAACT* (**A**) and *Δerg12* complemented with *GbMVK* (**B**). The strains were grown on YPD and YPG at 30 °C for 3 days with the exception of *Δerg10* with *GbAACT* and *Δerg12* with *GbMVK*.

The ergosterol synthesized from MVA pathway is essential for yeast survival [31,32]. A disruption of the MVA pathway genes is lethal in yeast [33,34]. To determine the function of *GbAACT* and *GbMVK*, two ergosterol auxotrophic strains of *Saccharomyces cerevisiae* that lacked the *AACT* or the *MVK* allele, named YPL028W (*ΔERG10*) and YMR208W (*ΔERG12*), respectively, were used for experiment. The pYES2 vectors, containing a yeast galactose-dependent promoter, were used as carrier for target genes in this study. The disrupted strains that harbored empty pYES2 could not grow on either the YPG expression medium or the YPD non-expression medium. Two expression vectors, namely, pYES2-GbAACT and pYES2-GbMVK, were constructed and transformed into strains YPL028W and YMR208W, respectively. YPL028W harbored pYES2-GbAACT, and YMR208W harbored pYES2-GbMVK, which grew well on the YPG medium. However, neither the YPL028W harbored with pYES2-GbAACT nor the YMR208W harbored with pYES2-GbMVK grew on the YPD medium (Figure 5). This proves that the transformed *GbAACT* can fix the functional loss of the *AACT* knockout yeast and that the transformed *GbMVK* can compensate the functional lack of the *MVK* knockout yeast. These results confirmed that GbAACT and GbMVK have AACT and MVK activity respectively.

### 2.5. Transcript Level of the Gene Expression Pattern of GbAACT and GbMVK in Different Tissues of G. biloba

**Figure 6:**
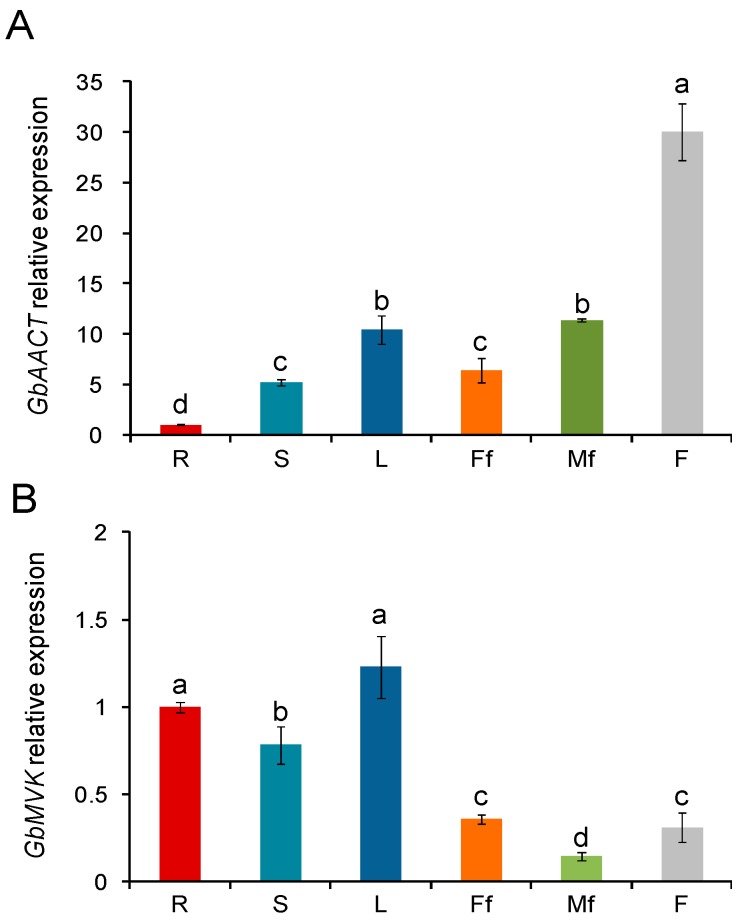
Gene expression Patterns of *GbAACT* (**A**) and *GbMVK* (**B**) in the various organs of *G. biloba* using qRT-PCR. The gene expression level of *GbAACT* and *GbMVK* in the root was set to 1, and those of *GbAACT* and *GbMVK* in other tissues were accordingly accounted and presented as the relative fold changes, respectively. R: root, S: stem, L: leaf, Ff: female flower, Mf: male flower, F: fruit. Data from qRT-PCR were shown as the mean ± SD (standard deviation) of three replicated assays. Means with different letters are significantly different at *p* < 0.05 by one-way ANOVA, with Tukey’s honestly significant difference test.

To present, reports corresponding to the *AACT* and *MVK* of *G. biloba* have been inexistent. To determine the expression patterns of *GbAACT* and *GbMVK* genes among different tissues in *G. biloba*, total RNA was extracted from roots, stems, leaves, female flowers, male flowers, and fruit, and cDNA was synthesized as mentioned above. qRT-PCR (Real-time quantitative reverse transcription PCR) was performed for *GbAACT* and *GbMVK* (Figure 6).

qRT-PCR revealed that the expression profile of *GbAACT* is distributed throughout all ginkgo tissues. Among different tissues, *GbAACT* had highest expression in fruit, followed by the leaf and the male flower. The *GbAACT* expression in the roots was significantly lower than that in the other tissues (Figure 6A). *AACT* genes were reported to be tissue-specific genes in other plants [27]. In *Bacopa monnieri*, the *BmAACT* gene was highly expressed in the root, followed by the stem and leaf [23], whereas results of a recent study in *Isodon rubescens* corresponded with this study in that *AACT* gene is more significantly expressed in leaves and flowers than in roots and stems [35]. Our data revealed that the transcript of *GbAACT* was detected in all ginkgo organs, including the root, stem, leaves, flowers, indeed overlapped with those of *GbLPS* (Levopimaradiene synthase) [20], *GbIDS* (1-Hydroxy-2-methyl-2-(*E*)-butenyl 4-diphosphate reductase) [19] and *GbMVD* (Mevalonate diphosphate decarboxylase) [11] in the roots and flowers. This result verifies the roots as the preferential site of TTL biosynthesis. However, one unexpected observation of this study is that the transcript level of *GbAACT* was higher in aerial tissues than roots. The possibility is that *GbAACT* in aerial parts of ginkgo is involved in the biosynthesis of yet to be identified terpenoids. 

As shown in Figure 6B, *GbMVK* was highly expressed in leaves, roots, and stems, and the highest *GbMVK* expression was detected in roots and leaves. The expression levels of *GbMVK* in floral organs and fruits were significantly low. A similar expression pattern of the *MVK* gene was found in *Panax notoginseng*, though in a lesser degree, because *PnMVK* is significantly expressed in roots, flowers, and leaves but is seldom expressed in stems [36]. The expression profile is consistent with the TTL distribution from earlier reports [37], showing that higher contents of TTLs were found in the leaves and roots than other tissues of ginkgo. A correlation exists between the transcript level of *GbMVK* and the content of TTLs, thereby suggesting that *GbMVK* plays an important role in the production of TTLs in ginkgo.

The biosynthesis organ of the TTL in *G. biloba* was not clearly reported, and long-distance transport was reported to be involved in the translocation of various compounds [38]. Therefore, considering the results of the present study and given that both *GbAACT* and *GbMVK* are highly expressed in the leaf and *GbMVK* is highly expressed in the root, we infer that the root and leaf could be most the important tissues for forming TTLs. To further determine which specific part of plant is involved in TTL biosynthesis, an isotopic tracer technique might be helpful.

### 2.6. Transcript Level of GbAACT and GbMVK and TTL Content Changes in Ginkgo biloba under the Induction of MeJA and SA Elicitors

**Figure 7:**
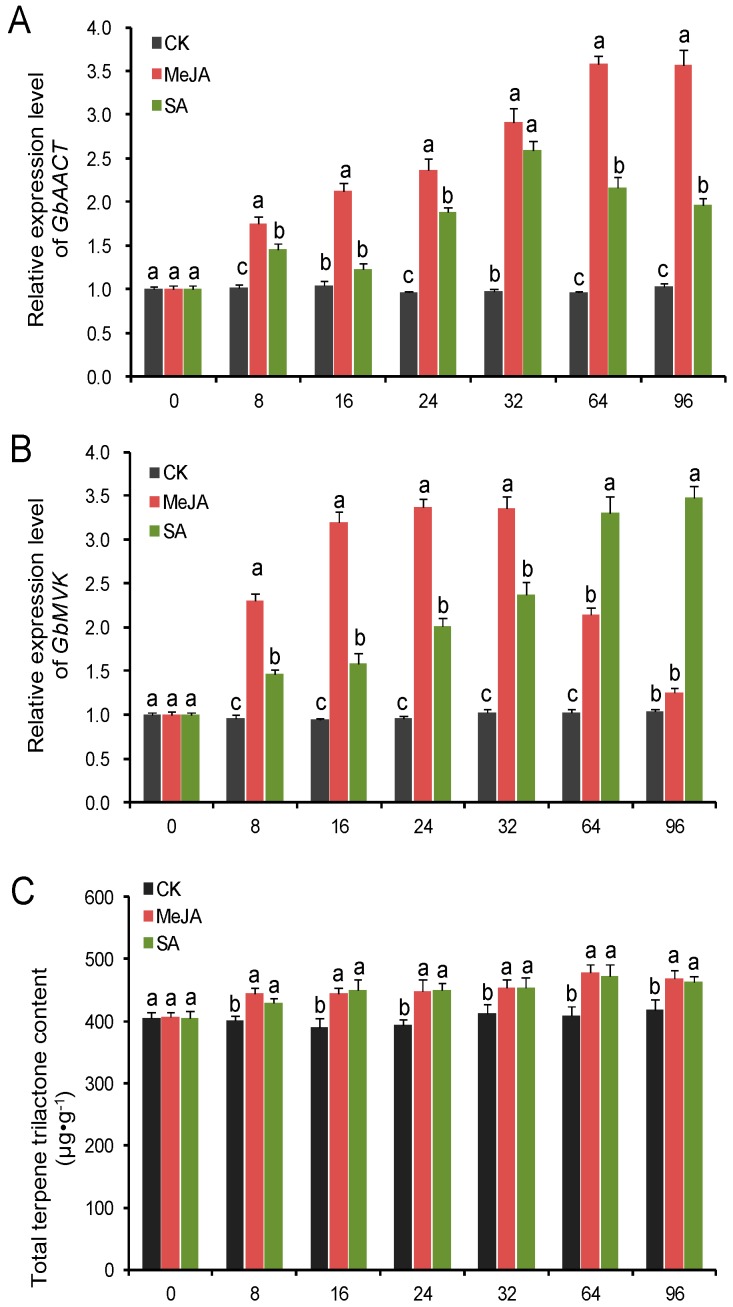
*GbAACT* and *GbMVK* expression level (**A**,**B**) and total terpene trilactone (TTL) content (**C**) changes in ginkgo by salicylic acid (SA) and methyl jasmonate (MeJA). The expression levels were normalized to the house-keeping gene *GAPDH* (glyceraldehyde-3-phospate dehydrogenase). Data from qRT-PCR were analyzed as expression ratios relative to the level of control (CK), and are shown as the mean ± SD of triplicate assays. Means with different letters from each time of post-treatments are significantly different at *p* < 0.05 by one-way ANOVA, with Tukey’s honestly significant difference test.

To understand the expression pattern of *GbAACT* and *GbMVK*, *GbAACT* and *GbMVK* transcript levels and TTL content were measured after seedlings at 4–5-leaf stage were treated with the elicitors MeJA and SA. qRT-PCR experiments clearly showed that SA and MeJA induction significantly increased *GbAACT* and *GbMVK* expression, and the TTL content was significantly enhanced after SA and MeJA treatments (Figure 7). The expression level of *GbAACT* continuously increased after MeJA treatment and peaked at 64 h with an 3.73-fold increase compared with that of control seedlings. The SA-treated seedlings showed the highest level of *GbAACT* expression at 32 h, and then slowly decreased (Figure 7A). With regard to the *GbMVK* gene, the expression level quckly increased after the seedlings were treated with MeJA for 8 h, and expression level of *GbMVK* gene peaked at 24 h after MeJA treatment. The *GbMVK* expression slightly changed from 16 h to 32 h, and the *GbMVK* expression decreased from 32 h to 96 h after treatment. Under SA treatment, *GbMVK* was highly expressed 8 h after the treatment, and *GbMVK* expression peaked until 96 h, with a 3.34-fold increase compared with that of the control (Figure 7B). TTL increased by 5.8% 8 h after SA treatment, and TTL content continuously increaesed from 0 h to 64 h. A similar increase was observed among MeJA-treated seedlings (Figure 7C).

With the regulation of SA and MeJA, plants were capable of adjusting to both abiotic and biotic stress [39]. SA and MeJA interact antagonistically aginst each other to induce transcription of defense-related genes by producing certain compunds and proteins [40,41,42,43]. Therefore, challenging ginkgo with SA and MeJA would activate defense-related genes in plants concomitantly increasing secondary metabolites. Indeed, the exogenous application of SA and MeJA has been performed to improve the biosynthesis of ginkgolide A and B and bilobalide in cell cultures of *G. biloba* [44]. In addition, some genes involved in TTL biosynthesis are upregulated after SA and MeJA treatments are administered in ginkgo. The transcript levels of *GbDXS* (1-deoxy-d-xylulose 5-phosphate synthase) [13], *GbCMK2* (4-(cytidine 5′-diphospho)-2-*C*-methyl-d-erythritol kinase) [15], *GbHMGR* [26], *GbIDS2* [45], and *GbMVD* [11] are induced by SA and MeJA and are positively involved in TTL biosynthesis in *G. biloba*. Similar to these reports, our data showed that the expression of *GbAACT* and *GbMVK* was upregulated and the TTL content was increased after SA and MeJA treatments, implying that the transcription of these genes responsible for TTL biosynthesis was enhanced. However, TTLs comprise a wide range of compounds and an integrated reaction of a cluster of genes related to TTL biosynthesis. The SA or MeJA-induced increase in TTL production in the present study may be arrtributed to an integrated effect on multiple genes related to TTL biosynthesis. Hence, it is interesting to unveil the expresssion profiling of other genes involved in TTL biosynthesis under SA or MeJA treatments in ginkgo, which will provide more insights into the regulatory role of SA and MeJA in TTL biosynthesis.

## 3. Experimental Section

### 3.1. Plant Materials

Plant samples were obtained from 18-year-old trees of *G. biloba* growing on the ginkgo garden of Yangtze University, Hubei, China (around N30.35, E112.14). The roots, stems, leaves, female and male flowers were collected in April 2014, and the fruits were collected in June 2014. Primer synthesis and DNA sequencing were performed by Sangon Biotechnology Company (Shanghai, China). Agarose Gel DNA Extraction Kit Ver. 4.0, pMD19-T vector kit, first-strand cDNA synthesis kit, dNTPs, RNasin, and *Taq* DNA polymerase were purchased from Takara Company (Dalian, China). A PrimeScript RT reagent kit with a gDNA (genomic DNA) Eraser and AceQ qPCR SYBR Green Master Mix were purchased from Vazyme Bio Inc. (Nanjing, China).

The *G. biloba* seedlings at 4–5-leaf stage were grown in a 24 ± 1 °C incubator with 100 μmol·m^−2^·s^−1^ light and a 16/8 h photoperiod. The seedlings were sprayed with 1 mM MeJA or 0.5 mM SA solution containing 0.1% (*v*/*v*) ethanol. Seedlings treated with 0.1% ethanol solution were used as control. The seedlings were harvested at 0, 8, 16, 24, 32, 64, and 96 h after treatment.

### 3.2. Cloning of the Full-Length cDNA of GbAACT and GbMVK

The RNA of *G. biloba* was extracted with TaKaRa MiniBEST Plant RNA Extraction Kit (Takara Bio Inc., Dalian, China) according to the manufacturer’s instruction. The extracted RNA was purified and used as templates to constructed RNA-seq library according to the manufacturer’s protocol. The transcriptome sequencing libraries were generated using a using a NEB Next^®^ UltraTM RNA Library Prep Kit for Illumina^®^ (NEB, Ipswich, UK). Sequencing run was performed at Biomarker Co., Beijing, China using Illumina Hiseq 2500 platform. In our previous work, we assigned the functional annotations of 35,113 unigenes [46]. The specific cDNA isolations were obtained through the PrimeScript first-strand cDNA synthesis kit. Primers were designed on the basis of the initial data of *AACT* and *MVK* unigenes in the transcriptome (GenBank accession numbers SRR3985386 and SRR3989536 for male and female strobilus, respectively). The sequences of all primers were shown Table 1. The PCR products were purified and ligated into pMD19-T vector (Takara Bio Inc., Dalian, China). The recombined plasmids were transformed into *E. coli* DH5α competent cells (Vazyme Bio Inc., Nanjing, China) and sequenced.

### 3.3. Bioinformatics Analysis and Molecular Evolution Analyses

Sequences were assembled and their ORF were analyzed with program Vector NTI 11.5.1 (Invitrogen, Paisley, UK,) [47]. Sequence comparison and amino acid translation were performed with DNAMAN 8.0 (Lynnon Biosoft, Quebec, QC, Canada). Protein isoelectric point (pI) and molecular weight were calculated via http://web.expasy.org/compute_pi/. The protein-conserved domain was analyzed via InterProScan [48] and http://www.ncbi.nlm.nih.gov. A phylogenetic tree of GbAACT and GbMVK from *G. biloba* and other plants was constructed through NJ method with CLUSTALX 2.0 (Conway Institute UCD Dublin, Dublin, Ireland) and MEGA 6.06 (Biodesign Institute, Tempe, AZ, USA) [49].

### 3.4. Construction of Expression Plasmids for Yeast Complementation

The coding regions of the *GbAACT* and *GbMVK* were amplified with two pairs of primers: GbAACT-yup and GbAACT-yud, GbMVK-yup and GbMVK-yud, respectively (Table 1). The forward primers contained the *Bam*HI restriction site, and the reverse primers contained the *Eco*RI restriction site. The amplified products and pYES2 vector (Invitrogen, Carlsbad, CA, USA) were digested with *Bam*HI and *Eco*RI, and then ligated into recombined vector pYES2-*GbAACT* and pYES2-*GbMVK*. PCR and sequencing were performed on positive clones. The constructed pYES2-*GbAACT* and pYES2-*GbMVK* plasmids were extracted and transformed into YPL028W (*ΔERG10*) and YMR208W (*ΔERG12*) with the Frozen-EZ Yeast Transformation II Kit (Zymo Research, Irvine, CA, USA). The wild-type *S*. *cerevisiae* strain YSC1021 is a diploid yeast, and *S. cerevisiae* strain *ΔERG10* lacking the *AACT* allele is the haploid yeast. The *S. cerevisiae* strain *ΔERG12* lacking the *MVK* allele is also a haploid yeast. The transformants were spotted on SC (-Ura) medium (6.7% yeast nitrogen base without amino acid, 2% galactose). The transformed diploid cells were induced to sporulate and subsequently formed haploid cells containing pYES2-*GbAACT* and pYES2-*GbMVK*. To further observe their growth condition, the diploid *S. cerevisiae* strain YSC1021 and transformed haploid strain YPL028W and YMR208W were separately grown on YPD (1% yeast extract, 2% Bacto Peptone, 2% glucose) and YPG (1% yeast extract, 2% Bacto Peptone, 2% galactose).

### 3.5. GbAACT and GbMVK Tissue-Specific Analysis

For quantification transcripts of *GbAACT* and *GbMVK* genes from *G. biloba* in different tissues, qRT-PCR was performed with primers designed (Table 1) using Primer premier 5.0 (Premier Biosoft International, Palo Alto, CA, USA). The PrimeScript RT reagent Kit was used with 100 ng of each total RNA to synthesize single-strand cDNA. The reaction was performed on MiniOpticon (Bio-Rad Laboratories, Inc., Alfred Nobel Drive Hercules, CA, USA) using SYBR Green detection with a reaction mixture (25 μL) containing 2× SYBR^®^ *Premix Ex Taq* II (Tli RNaseH Plus, Takara Bio Inc., Dalian, China), 0.4 μM each of forward and reverse primers, and 2 ng/μL of template cDNA. PCR amplification was performed under the following conditions: 95 °C for 30 s, followed by 40 cycles of 95 °C for 5 s and 60 °C for 30 s. All experiments were performed in triplicate, and the mean value was analyzed. Master mix without template was treated as negative control in each reaction. A glyceraldehyde-3-phospate dehydrogenase house-keeping gene (GAPDH) was used as internal control for normalization of all the reactions [50]. The primers used for the normalization (GbGAPDHF and GbGAPDHR) and expression analysis of *GbAACT* (GbAACT-qF and GbAACT-qR) and *GbMVK* (GbMVK-qF and GbMVK-qR) are shown in Table 1. All of the reactions were run in triplicate and repeated thrice. Gene expression analysis 2^−ΔΔCt^ method [51] was used to normalize the relative gene expression of the transcript in different tissue types.

### 3.6. Determination of TTL Contents in Ginkgo under Induction of MeJA and SA Elicitor

*G. biloba* seedlings were sampled and freeze-dried. Ginkgolide A, ginkgolide B, ginkgolide C, and bilobalide were extracted and quantified by gas chromatography with wide bore capillary column method [52] with minor modification. In detail, an appropriate amount of purified methanolic fraction was evaporated to dryness and trimethylsilylated by adding 100 μL of a silylating agent (Trisil BSA formula D, Sigma-Aldrich, Darmstadt, Germany). This mixture was vortex and heated for 2 h at 80 °C. Analysis by Gas chromatography-flame ionization detection (GC-FID) was performed with a Shimadzu GC-14B (Shimadzu, Japan) equipped with a 30 m × 0.53 mm × 1.5 μm TC-1 capillary column (HP-5, Agilent, San Francisco, CA, USA). The temperatures of the column, injector, and detector were maintained at 290 °C, 320 °C, and 320 °C, respectively. He was used as the carrier gas at a flow rate of 3.1 mL/min. TTL content was calculated as the sum of ginkgolide A, ginkgolide B, ginkgolide C, and bilobalide contents by dry weight percentages. Each sample was evaluated in triplicate, and data were represented as means ± SD (*n* = 3).

### 3.7. Statistical Analysis

Data were analyzed with using the statistical software SPSS 11.0 for Windows (SPSS Inc., Chicago, IL, USA). Comparisons between multiple treatment groups were performed using one-way ANOVA, with Tukey’s honestly significant difference test. *p* < 0.05 was considered to be statistically significant.

## 4. Conclusions

The genes *GbAACT* and *GbMVK* encoding AACT and MVK of the MVA pathway and the enzymes catalyzing the first and fourth steps in this pathway, respectively, were cloned and characterized. Bioinformatics analysis revealed that the deduced GbAACT and GbMVK harbored a highly similar identity to AACTs and MVKs of other plants. Functional complementation experiments in yeast demonstrated that *GbAACT* and *GbMVK* encode functional AACT and MVK, respectively. The transcript levels of *GbAACT* and *GbMVK* significantly increased in response to MeJA and SA treatments, and this increase corresponded to the increase in the TTL content after SA or MeJA treatment was performed. qRT-PCR analysis showed that *GbAACT* and *GbMVK* are tissue-specific genes. The transcript level of *GbAACT* is highly expressed in fruits, leaves, and floral organs, whereas the transcript level of *GbMVK* is highly expressed in leaves, roots, and stems. For further unveiling of the function of *GbAACT* and *GbMVK*, a plant expression vector containing the *GbAACT* or *GbMVK* has been constructed and a study of the genetic transformations of ginkgo is underway in order to investigate the potential role in enhancing TTL accumulation by genetic engineering.

## Figures and Tables

**Table 1:** Primers used in this study. ORF: open-reading frame.

Usage	Primer Name	Primer Sequence 5′-3′
ORF-PCR	GbAACT-F	ATGGGTTCTACATCAGGCTCAGATTTGTT
	GbAACT-R	TTACATGAGCTCCAAGACAACTGCAG
	GbMVK-F	ATATTGGGAGTTCAGAATGGTGC
	GbMVK-R	ACATTTATGAGCTTGAGCATTGGAA
qRT-PCR	GbAACT-qF	CATTGGGGGTCTGAGTGGTTC
	GbAACT-qR	CATTGTTGCTTTCATTCCCGA
	GbMVK-qF	GGCATTAGTTGCTGGAGTTTCTGAG
	GbMVK-qR	GGTGCCTGAATAATCTCTGCCATCT
	GbGAPDHF	CTGGCGTAGAGTATGTGGTTGAAT
	GbGAPDHR	CACGCCAACAACGAACATG
Yeast-PCR	GbAACT-yup	CGGGATCCATGGGTTCTACATCAGGCTCAG
	GbAACT-yud	CGGAATTCTTACATGAGCTCCAAGACAACTG
	GbMVK-yup	CGGGATCCATATTGGGAGTTCAGAATGGTGC
	GbMVK-yud	CGGAATTCACATTTATGAGCTTGAGCATTGGAA
